# Transmission of Hemagglutinin D222G Mutant Strain of Pandemic (H1N1) 2009 Virus

**DOI:** 10.3201/eid1605.091858

**Published:** 2010-05

**Authors:** Simona Puzelli, Marzia Facchini, Domenico Spagnolo, Maria A. De Marco, Laura Calzoletti, Alessandro Zanetti, Roberto Fumagalli, Maria L. Tanzi, Antonio Cassone, Giovanni Rezza, Isabella Donatelli, the Surveillance Group for Pandemic A (H1N1) 2009 Influenza Virus in Italy

**Affiliations:** National Institute of Health, Rome, Italy (S. Puzelli, M. Facchini, D. Spagnolo, M.A. De Marco, L. Calzoletti, A. Cassone, G. Rezza, I. Donatelli); Università degli Studi di Milano, Milan, Italy (A. Zanetti); Università degli Studi di Milano-Bicocca, Milan (R. Fumagalli); Università degli Studi di Parma, Parma, Italy (M.L. Tanzi); 1 Members of this group are listed at the end of this article.

**Keywords:** Influenza A virus, influenza, H1N1 subtype, pandemic, mutation, transmission, viruses, Italy, dispatch

## Abstract

A pandemic (H1N1) 2009 virus strain carrying the D222G mutation was identified in a severely ill man and was transmitted to a household contact. Only mild illness developed in the contact, despite his obesity and diabetes. The isolated virus reacted fully with an antiserum against the pandemic vaccine strain.

On November 20, 2009, the Norwegian Institute of Public Health reported to the World Health Organization a mutation in the hemagglutinin (HA) of pandemic (H1N1) 2009 virus, consisting in a change of aspartic acid (D) with glycine (G) at aa 222. The mutation had been detected in 3 patients (the first 2 fatal cases in the country and in 1 patient with severe pneumonia) among ≈70 other patients with pandemic (H1N1) 2009, suggesting that it was not widespread in Norway (*1*). The same mutation has been also detected in Brazil, China, Japan, Mexico, Ukraine, the United States, France, and Spain (*1**,**2*).

The D-to-G mutation among the 1918 influenza virus variants (*3*) correlated with a shift from α2-6–linked sialic acid preference to a dual α2-3/α2-6 specificity. However, whether such a mutation may alter receptor binding specificity in the pandemic (H1N1) 2009 virus is unknown.

Although several pandemic (H1N1) 2009 viral strains sharing this mutation were detected in fatal cases, the same mutation also was detected in some mild cases; conversely, viruses from numerous fatal cases have not shown the same mutation. Thus, the clinical and public health significance of this finding remains unclear. The mutation appears to occur sporadically and spontaneously. No links between the small number of patients infected with the mutated virus have been found, and the mutation did not appear to spread (*4*). On the basis of results from a retrospective HA1 sequence analysis performed on pandemic (H1N1) 2009 viral isolates in Italy, we report on a transmission event of this virus carrying the D222G mutation.

## The Study

We reexamined the HA sequences of 130 influenza A (H1N1) virus strains identified from patients affected by pandemic (H1N1) 2009. The neuraminidase sequences of some of these viruses also have been analyzed.

All 130 strains had been obtained from clinical samples (nasal, pharyngeal, or nasopharyngeal swabs and/or tracheal aspirates) collected during May–November 2009 in the context of virologic surveillance conducted by the National Influenza Centre, in collaboration with the regional laboratory network. These samples were obtained to study the evolution of the pandemic strain. Forty-one HA gene sequences examined in the present study were retrieved from the National Center for Biotechnology Information, 3 from the Global Initiative on Sharing Avian Influenza Data database, and 86 HA sequences (47 directly from the clinical samples and 39 from cell culture supernatant) were obtained at the National Influenza Centre (NIC) with the following procedure. Viral RNAs were extracted by using the QIAamp Viral RNA Mini Kit (QIAGEN, Santa Clara, CA, USA) and amplified by reverse transcription–PCR (RT-PCR) (*5*). HA amplicons were sequenced by using the BigDye Terminator Cycle-Sequencing Ready Reaction (Applied Biosystems, Foster City, CA, USA) and ABI Prism 310 DNA sequencer (Applied Biosystems). All the NIC sequences were deposited in the GenBank database under the accession numbers reported in the Figure. Sequences were assembled and aligned using Lasergene package, version 4.0 (DNASTAR, Madison, WI, USA). BioEdit software version 4.0 (www.mbio.ncsu.edu/BioEdit/bioedit.html) of the MEGA software package (www.megasoftware.net) was used to estimate phylogenies from the nucleotide sequences and to construct phylogenetic trees by using the neighbor-joining algorithm and maximum-likelihood method.

Among 130 patients, 23 of whom had severe disease (i.e., requiring hospitalization), only 1 was infected with a virus showing the D222G change. The patient, a man 25 years of age from northern Italy, had a febrile illness on August 17. One week later, he was admitted to an intensive care unit with severe pneumonia and acute respiratory distress syndrome, which resolved after treatment with extracorporeal membrane oxygenation. Influenza A (H1N1) viral genome showing the D222G mutation was identified through direct sequencing of nasopharyngeal swab and tracheal aspirate collected on August 27. No sequence could be retrieved from a nasal wash, which was obtained the same day and was positive in real-time RT–PCR for pandemic (H1N1) 2009 virus. No viral growth was detected in MDCK cells seeded with both clinical samples.

To identify possible transmission chains of the mutated virus, we analyzed the genome of the viral strain detected in the throat swab of the father of the index case-patient, 55 years of age, who was obese and had diabetes. He became moderately ill on August 25 but did not require hospitalization or antiviral treatment. The virus isolated in MDCK cells from the sample obtained on August 27 had the same HA mutation, D222G. Viral strains from index and contact cases were susceptible to oseltamivir, as determined by lack of the specific oseltamivir-resistance marker (His274Tyr, N2 numbering) in neuraminidase sequences, which were identical in both viral strains. Additional samples analyzed from close contacts (i.e., 4 healthcare workers, 4 family members, and 2 friends) of the 2 patients all were negative for pandemic (H1N1) 2009 virus.

Comparison of the 2 HA1 sequences from the index case-patient and his father showed an additional substitution in the latter (G155E), which is located close to the receptor-binding pocket. Furthermore, sequence analyses showed that all virus strains containing a change in HA1 position 222 (D222G or D222E) also showed a second substitution in position 203 (D203T), when compared with the A/California/7/2009 vaccine strain (Figure). However, the effect of this second mutation on the HA receptor-binding properties is still unclear. The HA1 genes from the index and contact case-patients shared 2 additional nucleotide changes, 1 synonymous substitution (T504C, N1 numbering) not found in any other sequence analyzed in the present study and another nucleotide change resulting in an amino acid substitution (P297S, N1 numbering), detected only in a small number of sequences from Italy. Hemagglutination-inhibition test of the isolated virus did not show substantial reduction in its reactivity with an antiserum against pandemic (H1N1) 2009 vaccine, as compared with the reactivity of an influenza virus A/California/7/2009 strain not carrying the G155E mutation (Table).

**Figure:**
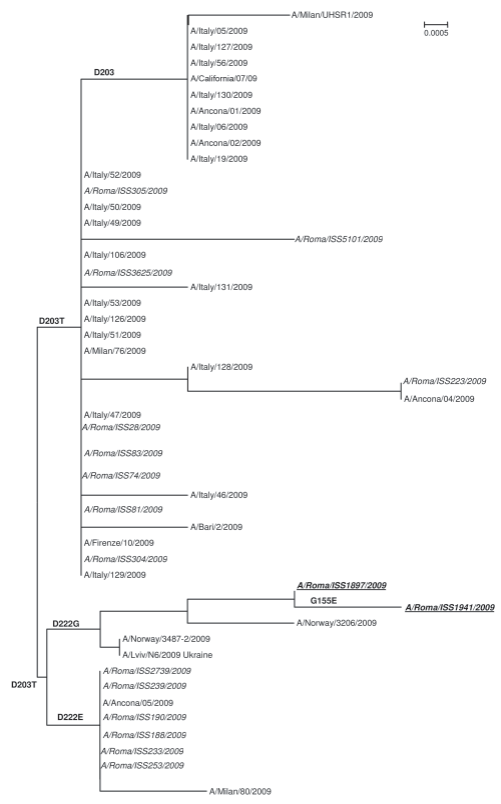
Phylogenetic relationships of hemagglutinin (HA) 1 sequences of pandemic (H1N1) 2009 viruses in Italy obtained from the National Influenza Centre (NIC)–Istituto Superiore di Sanità (ISS) and the National Center for Biotechnology Information. The NIC-ISS sequences are in *italics*. Isolates from the index case-patient and his father are indicated in **boldface**. Amino acid mutations of interest in this study are reported on the nodes. All the NIC sequences obtained in the present study were deposited in the GenBank database under the following accession numbers: (hemagglutinin sequences) GU451262–GU451280; GU576500–GU576502; GU576504; GU576506; GU576508; GU576510; GU576512; GU576514-GU57615; GU576517; GU576519; GU576521-GU576522; GU576524; GU576526-GU576527; GU576529; GU576531-GU576532; GU576534; GU576536-GU576540; GU585403–GU585443; (neuraminidase sequences) GU936490-GU936491. Scale bar indicates proportion of nucleotide substitutions per site.

**Table:** Hemagglutination-inhibition test results of pandemic (H1N1) 2009 viruses, Italy

Viruses	Postinfection ferret serum titer against	
	A/Brisbane/59/2007 seasonal H1N1	A/California/7/2009 pandemic H1N1
A/Brisbane/59/2007 (H1N1)	1,280	<10
A/California/7/2009 (H1N1v)	<10	1,280
Father’s virus isolate (D222G + G155E)	<10	640
A/Rome/ISS11/2009	<10	320

## Conclusions

Identification of a pandemic (H1N1) 2009 virus strain carrying the D222G mutation and its association with the first fatal cases of influenza in Norway raised some concern about emergence of a viral strain with increased pathogenicity. No data have been reported on transmission capacity of this and other D222G variants, occasionally identified worldwide. Our findings suggest that the D222G mutated virus is to some extent transmissible. However, a number of close contacts were identified who did not acquire the infection.

Whether the mutated virus may have a lower fitness for receptors in the high respiratory tract, which may affect transmission, remains undefined. Furthermore, the mutation, which was first found in a severely affected man, was transmitted to a family member (father), who had only a mild illness, despite risk for severe infection. The HA1 sequence from this latter case presented an additional amino acid substitution (G155E), which was not found in any other sequence analyzed in our study and rarely detected among all sequences available from GenBank. Recent data suggest that amino acid substitution at this position may be critical for the switch from dual α2-3/α2-6 binding specificity to α2-6 linkage (*6*), which is preferentially recognized by human influenza viruses and expressed mainly in the upper respiratory tract. This change might be partially responsible for the milder influenza illness developed by the father of the index case-patient. In addition, the G155E mutation in laboratory-generated variants of pandemic (H1N1) 2009 virus has been associated with loss of antigenicity (*7*). In our study, the natural isolates of the virus carrying both the G155E and the D222G mutations had comparable antigenicity with the A/California/7/09-vaccine strain. Finally, our data do not support the association of the D222G mutation with severe disease.
